# Acceleration of Bone Repair in NOD/SCID Mice by Human Monoosteophils, Novel LL-37-Activated Monocytes

**DOI:** 10.1371/journal.pone.0067649

**Published:** 2013-07-03

**Authors:** Zhifang Zhang, John E. Shively

**Affiliations:** Department of Immunology, Beckman Research Institute of City of Hope, Duarte, California, United States of America; Montana State University, United States of America

## Abstract

**Background:**

An incomplete understanding of bone forming cells during wound healing and ectopic calcification has led to a search for circulating cells that may fulfill this function. Previously, we showed that monoosteophils, a novel lineage of calcifying/bone-forming cells generated by treatment of monocytes with the natural peptide LL-37, are candidates. In this study, we have analyzed their gene expression profile and bone repair function.

**Methods and Findings:**

Human monoosteophils can be distinguished from monocytes, macrophages and osteoclasts by their unique up-regulation of integrin α3 and down-regulation of CD14 and CD16. Monoosteophils express high mRNA and protein levels of SPP1 (osteopontin), GPNMB (osteoactivin), CHI3L1 (cartilage glycoprotein-39), CHIT1 (Chitinase 1), MMP-7, CCL22 and MAPK13 (p38MAPKδ). Monocytes from wild type, but not *MAPK13* KO mice are also capable of monoosteophil differentiation, suggesting that MAPK13 regulates this process. When human monoosteophils were implanted in a freshly drilled hole in mid-diaphyseal femurs of NOD/SCID mice, significant bone repair required only 14 days compared to at least 24 days in control treated injuries.

**Conclusion:**

Human derived monoosteophils, characterized as CD45^+^α3^+^α3β^+^CD34^−^CD14^−^BAP (bone alkaline phosphatase)^−^ cells, can function in an animal model of bone injury.

## Introduction

Bone generation, maintenance and repair involve osteoblasts, osteoclasts, and osteocytes. Osteoblasts are cells of mesenchymal stem cell (MSC) origin that secrete bone-matrix proteins and promote mineralization. Differentiated osteoblasts embedded in the bone matrix or osteocytes have an as-yet unclear role in mechanotransduction [1]. Osteoclasts are cells of hematopoietic origin that decalcify and degrade the bone matrix by acid decalcification and proteolytic degradation. They are large, multinucleated cells formed by the fusion of precursor cells of the monocyte–macrophage lineage. Osteoclasts may be generated in vitro by treatment of monocytes with RANKL and M-CSF [2], [3].

An incomplete understanding of bone forming cells has led to an unfilled clinical need including healing of nonunion of bone fractures and prevention of ectopic calcification [4], [5]. Since wound healing involves the recruitment of circulating immune cells to the injured site and bone marrow has a unique structural relationship with broken bone, immune cells have been proposed to play a role in bone repair [6]. Several CD34^+^ cell populations derived from peripheral blood with a hematopoietic lineage phenotype have been shown to have the potential to differentiate to osteoblast-like cells, such as endothelial progenitor cells (EPCs) [7], monocyte-derived mesenchymal progenitors (MOMPs) [8], circulating osteoblast-lineage cells (COCs) [9], fibrocytes [10], and circulating osteogenic precursor (COP) cells [11].

Two recent reports directly implicated purified peripheral blood monocytes as potential calcifying cells in ectopic bone formation [12] and vascular calcification [13]. First, Fadini et al. [13] showed that a subpopulation of monocytes (CD45^+^CD14^+^CD68^+^CD34^−^) that expressed cell surface osteocalcin and bone alkaline phosphatase (BAP), termed myeloid calcifying cells (MCC), formed spotty calcifications when implanted in Matrigel plugs in nude mice. Second, we demonstrated that monoosteophils (CD45^+^CD34^−^CD14^−^CD16^−^CD90^−^BAP^−^), derived from LL-37 treated human CD14^+^ monocytes, express some characteristic proteins of both osteoblasts and osteoclasts and are able to form bone in both in vitro and in vivo models [12]. Since LL-37 is produced by keratinocytes, granulocytes and macrophages during inflammation and recruits monocytes [14]–[16], it is possible that the recruited monocytes undergo LL-37 induced differentiation into monoosteophils, which in turn initiate bone repair.

Therefore, we investigated the gene expression profile and bone repair function of monoosteophils. Here, we show that monoosteophils can be distinguished from monocytes, macrophages and osteoclasts by their unique up-regulation of integrin α3. Monoosteophils express high mRNA and protein levels of SPP1 (osteopontin), GPNMB (osteoactivin), CHI3L1 (cartilage glycoprotein-39), CHIT1 (Chitinase 1), MMP-7, CCL22 and MAPK13 (p38MAPKδ). Monocytes from wild type but not *MAPK13* KO mice were able to differentiate into monoosteophils upon LL-37 treatment, suggesting that MAPK13 is a key signal molecule for the differentiation of monoosteophils. When human monoosteophils were implanted in a freshly drilled hole in mid-diaphyseal femurs of NOD/SCID mice, significant bone repair required only 14 days compared to at least 24 days in control treated injuries. Thus, human derived monoosteophils, characterized as CD45^+^α3^+^α3β1^+^CD34^−^CD14^−^BAP^−^ cells, can function in an animal model of bone injury.

## Results

### SEM/EDS Analysis of Monoosteophil-formed Nodules

**Figure 1:**
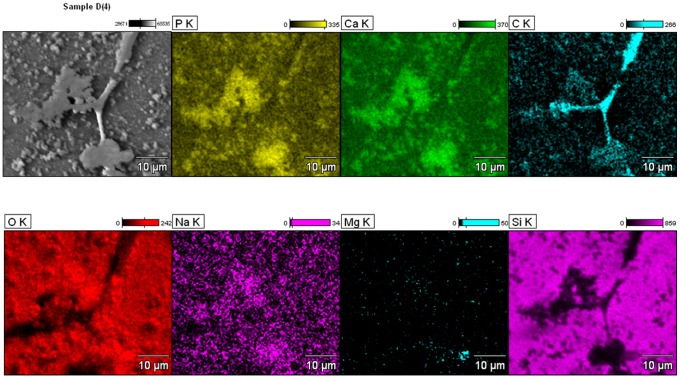
Built-up structures of LL-37-differentiated monocytes (monoosteophils) on BioCoat™ Osteologic™ Discs consists of phosphorus, calcium, carbon, and sodium. Monocytes were incubated with 5 µM LL-37 on BioCoat™ Osteologic™ Discs in 5% CO_2_ atmosphere for 7 weeks, built-up structures and cells were identified by FEI Nova NanoSEM 230 Field Emission SEM and the Thermo NORAN System 7 EDS analyzer. Elements of built-up structure were shown in the figure. P = phosphorus, Ca = calcium, C = carbon, O = oxygen, Na = sodium, Mg = magnesium, Si = silicon.

We previously showed that monoosteophils built refractive, raised granules on the surface of osteologic discs that were identified as inorganic phosphate deposits by von Kossa staining [12]. In order to verify that the deposits were mineralized calcium phosphate, we used scanning electron microscopy (SEM) coupled with an energy dispersive X-ray spectroscopy (EDS) analyzer to investigate the elemental composition of the granules. As shown in **Figure 1** **,** the deposits contained phosphorus, calcium, carbon and sodium, demonstrating that monoosteophils produce deposits of typical calcifying/bone-forming cells.

### Differentiation Markers and Proliferative Capacity of Monoosteophils

**Figure 2:**
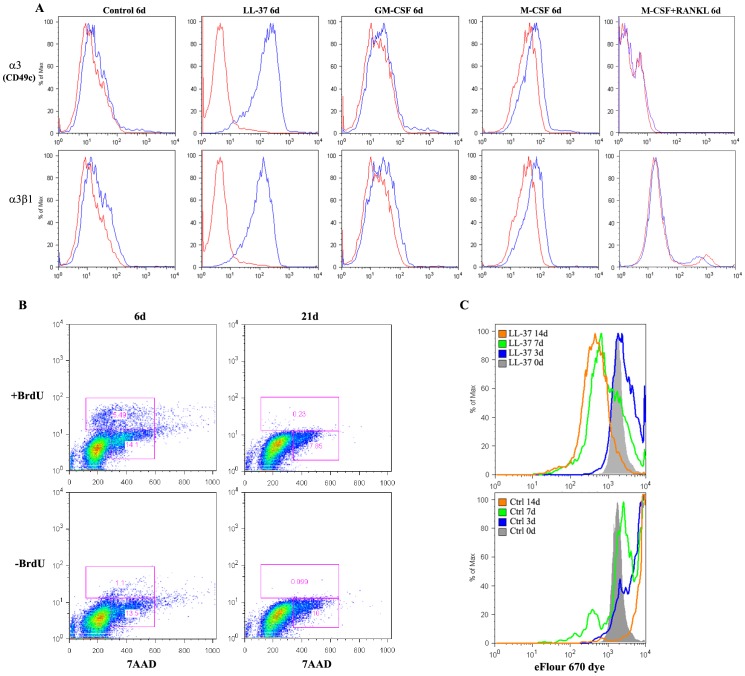
Differentiation markers and proliferative capacity of monoosteophils. **A.** Monocytes were incubated in the absence or presence of LL-37 (5 µM), GM-CSF (20 ng/mL), M-CSF (50 ng/mL), or M-CSF and RANKL (both at 25 ng/mL) for 6 days. Surface staining of Integrin α3 and α3β1 were analyzed using flow cytometry**. B–C.** Monocytes were incubated in the presence of 5 µM LL-37 for different days and proliferative capacity was detected using either (**B**) FITC BrdU/7AAD flow kit or (**C**) Cell proliferation dye eFluor 670. Data shown were from three independent experiments.

Monoosteophils become strongly adherent 6 days after a single treatment with 5 µM LL-37. Cell surface staining with a panel of anti-integrin antibodies (**Figure S1**) showed a unique expression of integrin α3 (CD49c) and the complex α3β1 compared to the lack of expression of integrin α3 on in vitro differentiated macrophages and osteoclasts (**Figure 2A**). Monoosteophils express high levels of integrin α6 (CD49e), while macrophages and osteoclasts express low levels (**Figure S1**). Ten percent of the cells were positive for integrin α3 at day 1, with a significant increase by day 3 after treatment with LL-37 (**Figure S2**). Integrin α3β1 is a cell surface receptor for collagen I, laminin, epiligrin, fibronectin, and entactin [17], all key ligands likely required for recruitment of these cells to a site of injury. Importantly, these cells were negative for surface scavenger receptor CD68 (**Figure S3A**), osteocalcin and BAP [12] distinguishing them from previously described COCs, MCCs, MOMPs, as well as osteoblasts and osteocytes.

The differentiation of LL-37 treated monocytes to monoosteophils takes place in two stages. Over the first 24 h there is a significant decrease in cell number, followed by an increase in cell number from day 2 to day 6, while the cell numbers of untreated monocytes continually decrease over the 6d period (data not shown). To determine the proliferative capacity of the monoosteophils, the cells were labeled with BrdU/7AAD or the Cell Proliferative Dye eFluor 670. Approximately 4% of the cells were BrdU positive vs 14% 7AAD positive at day 6, declining to <1% BrdU positive at day 21 (**Figure 2B**). Using cell proliferation dye eFluor 670, we found that monoosteophils synchronously divided at day 7 and day 14 with no preference for a subset of the cells (**Figure 2C**). Although control monocytes showed an accumulation of proliferation dye eFluor 670, microscopic examination revealed that a portion differentiated into macrophages and ingested the majority of apoptotic cells. Interestingly, when the monocytes were labeled with the classic proliferative dye CFSE, all of the cells died in both the untreated controls and LL-37 treated monocytes (data not shown). Taken together, these results demonstrate that monoosteophils have a limited capacity for proliferation, and together with our previous study [12], show that they survive for extended periods, up to one year in culture.

### Gene Expression Analysis of Monoosteophils

**Figure 3:**
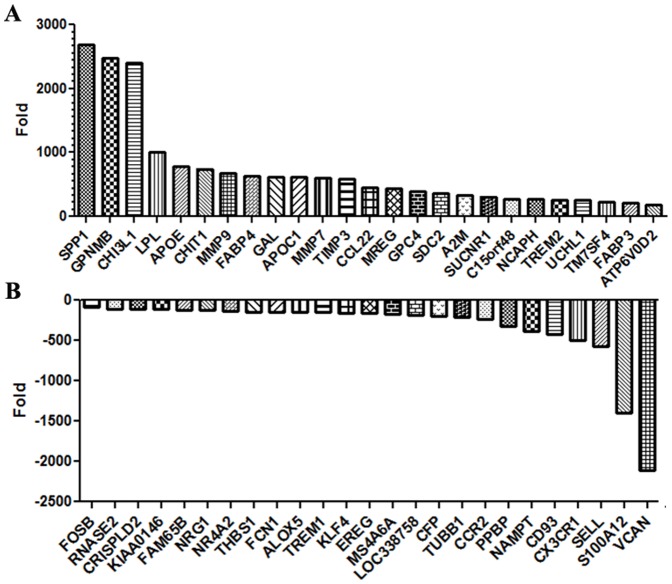
Microarray analysis of monoosteophils. Gene expression profiles of 6 day monoosteophils in comparison with freshly isolated monocytes were performed using Affymetrix gene chip analysis: top 50 up-regulated (**A**) and down-regulated (**B**) were shown as fold change.

Since the monoosteophil is a novel type of calcifying/bone forming cell and shares some characteristics with osteoblasts that rely on bone morphogenic proteins (BMPs) for differentiation from their precursors, we analyzed their gene expression profile using a focused human BMP pathway array. In a comparison of day 6 monoosteophils with freshly isolated monocytes (day 0), the most dramatic up-regulated gene was MAPK13 (p38MAPKδ), while most other important BMP pathway genes, such as Smad1-5, BMP2, BMP4, FOS, ATFs, and p38MAPKα/β/γ were down-regulated or unchanged (**Table S1**). Since this analysis suggested that monoosteophils utilize a pathway distinct from the BMP pathway, the more comprehensive Affymetrix gene chip analysis was employed. In this analysis over 3,977 genes were found to be differentially expressed between the two groups (p<0.05), of which 2,146 genes were up-regulated and 1,831 genes were down-regulated. Genes unique to monoosteophils as well as found in osteoblasts, osteocytes or osteoclasts are summarized in **Table S2**. The 50 top scoring up-regulated and down-regulated genes are shown in **Figure 3**. Not surprisingly, the top 2 up-regulated genes are bone formation related genes, *SPP1* (2,694 fold) or osteopontin and *GPNMB* (2,477 fold) or osteoactivin. Osteopontin, initially described as a major noncollagenous protein in bone (also called bone sialoprotein I), is known to be involved in bone remodeling, wound repair, immune function, angiogenesis, and cell survival [18]. Osteoactivin, first cloned and identified from osteoblasts [19], has been characterized as a downstream mediator of BMP-2-induced osteoblast differentiation [20] and is associated with significant bone fill in the rat critical-size calvarial through-and-through defect [21]. Another highly up-regulated gene, *CHI3L1* (2,409 fold) or cartilage glycoprotein-39, a catalytically inactive member of glycohydrolase family 18, is a 39-kDa glycoprotein secreted by articular chondrocytes [22], synoviocytes [23], and macrophages [24] and its expression has been linked to both rheumatoid arthritis and osteoarthritis [25]. Within the top 13 up-regulated genes, *MMP-9* (680 fold), *MMP-7* (600 fold) and *TIMP3* (TIMP metallopeptidase inhibitor 3, 584 fold) are also involved in bone metabolism [26]. Interestingly, *LPL* (lipoprotein lipase, 1,001 fold), *APOE* (apolipoprotein E, 778 fold), *FABP4* (fatty acid binding protein 4, 680 fold), and *APOC1* (apolipoprotein C–I, 614 fold) are atherosclerosis related genes in which activated monocytes play a role [27]. Furthermore, it was recently reported that cathelicidins (CRAMP in mouse, LL-37 in human) promote atherosclerosis by enhancement of the recruitment of inflammatory monocytes [28]. In this respect, the up-regulation of *CCL22* (macrophage-derived chemokine, 452 fold) by monoosteophils may be responsible for monocyte recruitment, just as LL-37 is also a chemotactic factor for monocytes. Another up-regulated gene of interest is *CHIT1* (743 fold, chitinase 1), elevated in Gaucher disease, atherosclerosis and tuberculosis, suggesting unanticipated roles in bone repair. In accordance with the BMP pathway array results, the top signal pathway gene was *MAPK13* (106 fold, **Table S2**). In terms of top down-regulated genes, *VCAN* (−2,104 fold, versican) stands out for its normal expression in chondrocytes [29]. The lack of its expression, suggests that monoosteophils do not express chondroitin sulfate, suggesting a negative role for this proteoglycan in bone repair. Taken together, these analyses not only distinguish monoosteophils from classic bone remodeling cells, but reveal connections to new pathways of bone repair and pathological conditions such as atherosclerosis that can involve calcification of tissues.

### Monoosteophils Secrete High Amounts of Osteopontin, Osteoactivin, Cartilage Glycoprotein-39, Chitinase 1, MMP-7 and CCL22

**Figure 4:**
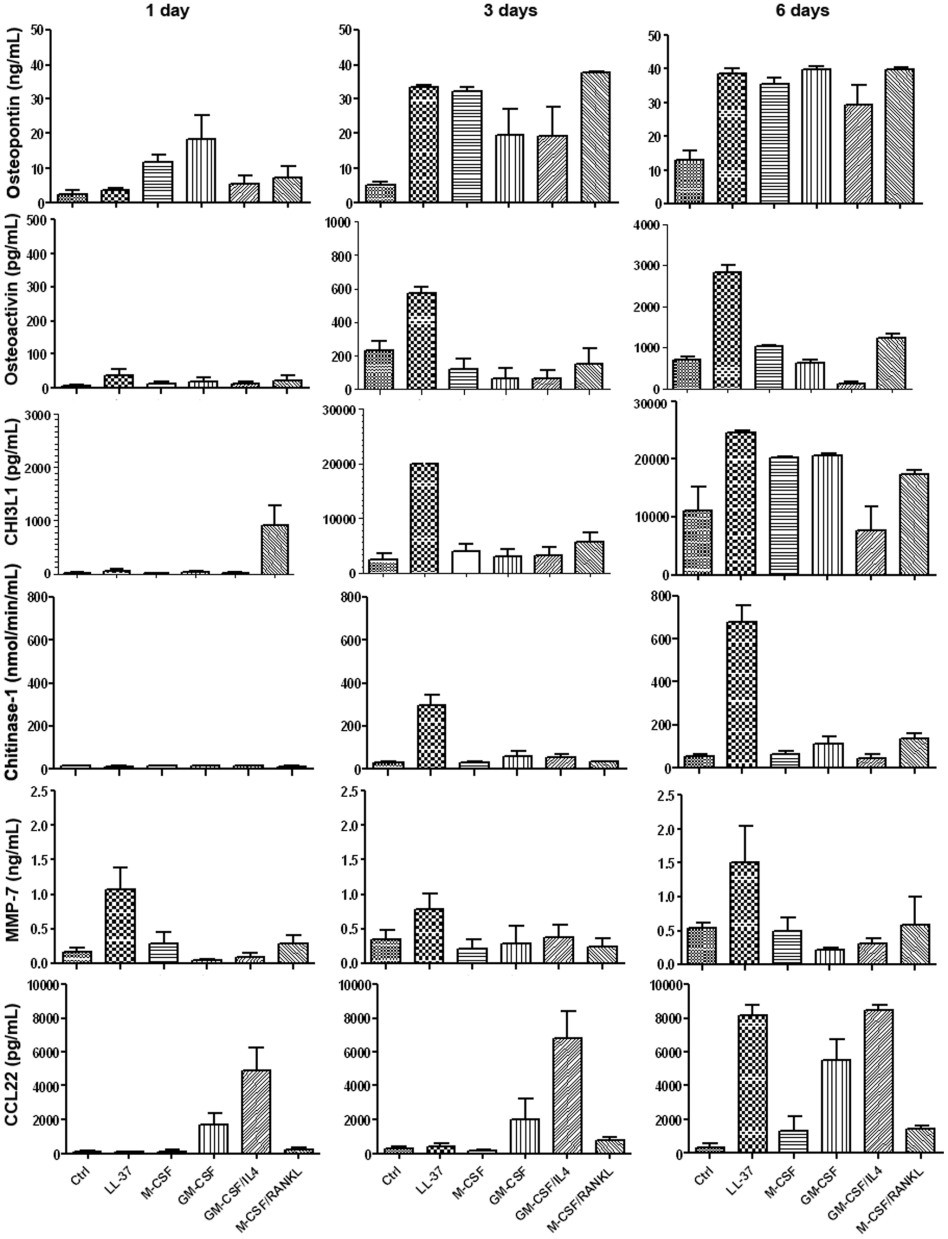
Monoosteophils release high level of osteopontin, osteoactivin, cartilage glycoprotein-39, chitinase 1, MMP-7 and CCL22. Monocytes were incubated in the absence or presence of LL-37 (5 µM), GM-CSF (20 ng/mL), GM-CSF+IL-4 (both 20 ng/mL), M-CSF (50 ng/mL) or M-CSF +RANKL (both at 25 ng/mL) for 1, 3 or 6 days. Supernatants were harvested and proteins were detected by using ELISA Kits (n = 3). CHI3L1: cartilage glycoprotein-39.

To confirm gene expression result at the protein level, we quantified the concentration of osteopontin, osteoactivin, cartilage glycoprotein-39, chitinase 1, MMP-7, and CCL22 in cultured supernatants of monocytes treated in a variety of ways at day 1, 3, and 6. As shown in **Figure 4**, all analyzed proteins of LL-37 treated monocytes (monoosteophils) are significant higher than untreated controls at day 6, and levels of osteoactivin, cartilage glycoprotein-39, chitinase 1, and MMP-7 were uniquely elevated in comparison with GM-CSF treated, M-CSF treated, GM-CSF+IL-4 treated (DCs) or M-CSF+RANKL treated (osteoclasts) monocytes at both day 3 and day 6. At day 1, osteoactivin and MMP-7 were highest in monoosteophils compared to the other monocyte-differentiated cell types. CCL22, the top chemokine hit in the gene chip analysis (**Figure 3**), significantly increased at day 6, but was low at day 1 and 3; however, this chemokine is not unique to monoosteophils, with expression also seen in GM-Mac and M-Mac (**Figure 4**). The receptor for CCL22, CCR4, was low at day 6 compared to GM-Mac and M-Mac (**Figure S3B**), suggesting that expression of its self ligand CCL22 was responsible.

### MAPK13 Signaling Pathway Plays Key Role in the Differentiation of Monoosteophils

**Figure 5:**
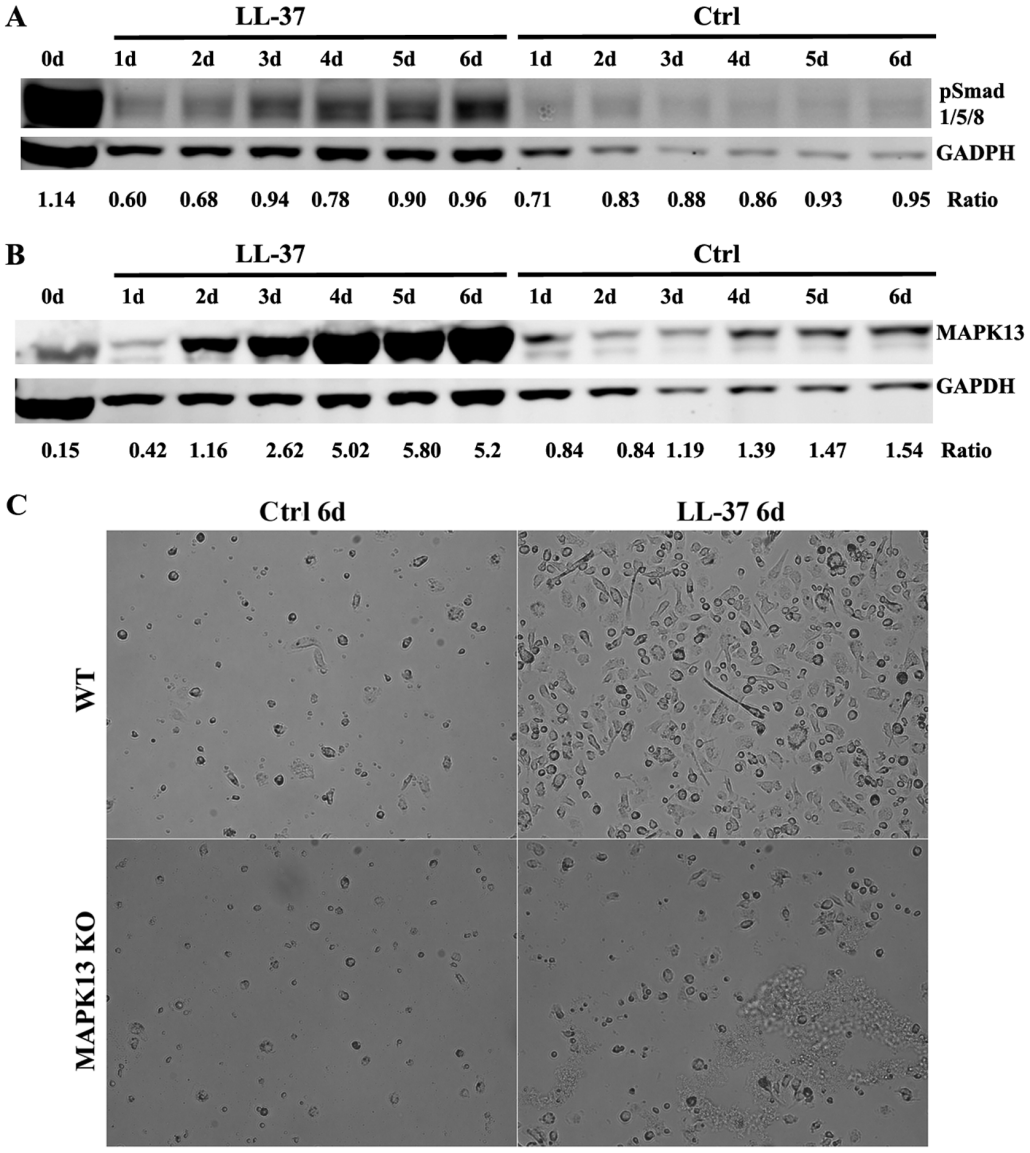
pSmad1/5/8 and MAPK13 signaling in the differentiation of monoosteophils. Human monocytes were incubated in the absence or presence of LL-37 (5 µM). Cells were harvested on days 0–6 and pSmad1/5/8 (**A**) and MAPK13 (**B**) were analyzed by western blot. (**C**) Mouse monocytes were isolated from bone marrow of *MAPK13* KO or wild type mice, cultured at the concentration of 1×10^6^ cells/mL in absence or presence of 5 µM CRAMP (murine LL-37) for 6 days, and observed using phase contrast microscopy (magnification, 200×). Data shown are representative of three independent experiments.

Osteoblasts and osteoclasts, among the most studied resident bone cells, critically rely on signaling pathways involving ERK, p38MAPKα/β/γ and Smads [30], [31]. Phospho-p38MAPKα/β/γ, phospho-ERK42/44, RunX2 and osterix were either low or unchanged in LL-37 treated monocytes at day 6 compared to untreated controls (**Figure S4A–D**). Time course analysis of phospho-Smad1/5/8 is especially interesting in that freshly isolated monocytes (0 d) are highly activated in this regard and lose this marker as soon as 1 d in both untreated and LL-37 treated cells (**Figure 5A**). In the case of monoosteophils, there is a slow increase over time for phospho-Smad1/5/8, suggesting a potential role for this signaling pathway. In accordance with our gene chip analysis, MAPK13 was dramatically up-regulated from 0–6 d during monoosteophil differentiation (**Figure 5B**). A time course of the expression of MAPK13 and 6 other genes potentially associated with MAPK13 showed that two of the genes (CHI3L1 and Chitinase 1) followed the same kinetics as MAPK13, suggesting that their expression profiles were related (**Figure S5**). Due to lack of reagents, we were unable to measure the degree of activation of MAPK13, but given its unique up-regulation, it is likely activated. Given the potential role of MAPK13 in human monoosteophil differentiation, we directly assessed its role in the differentiation of mouse monocytes into monoosteophils. Treatment of murine monocytes from wild type but not *MAPK13* KO mice with CRAMP (murine LL-37) was able to differentiate these cells with an identical morphology and survival characteristics to authentic human monosteophils (**Figure 5C**). Analysis of the cell surface markers of CRAMP vs GM-CSF treated murine monocytes for α3 integrin, α6 integrin, CD68 and alkaline phosphatase revealed up-regulation of both α3 and α6 integrins, characteristics shared with LL-37 treated human monocytes (**Figure S6**).

In other studies, MAPK13 is up-regulated in arterial smooth muscle cells undergoing calcification [32], a process likely involving monocytes in an inflammatory milieu. MAPK13 null mice exhibit improved glucose tolerance due to enhanced insulin secretion from pancreatic β cells and are protected against high-fat-feeding-induced insulin resistance and oxidative stress-mediated β cell failure [33]. Thus, MAPK13 may play multiple roles in signal transduction, depending on its tissue expression.

### Monoosteophils Accelerate Cortical Bone Repair in a Drilled Hole Bone Defect Model

**Figure 6:**
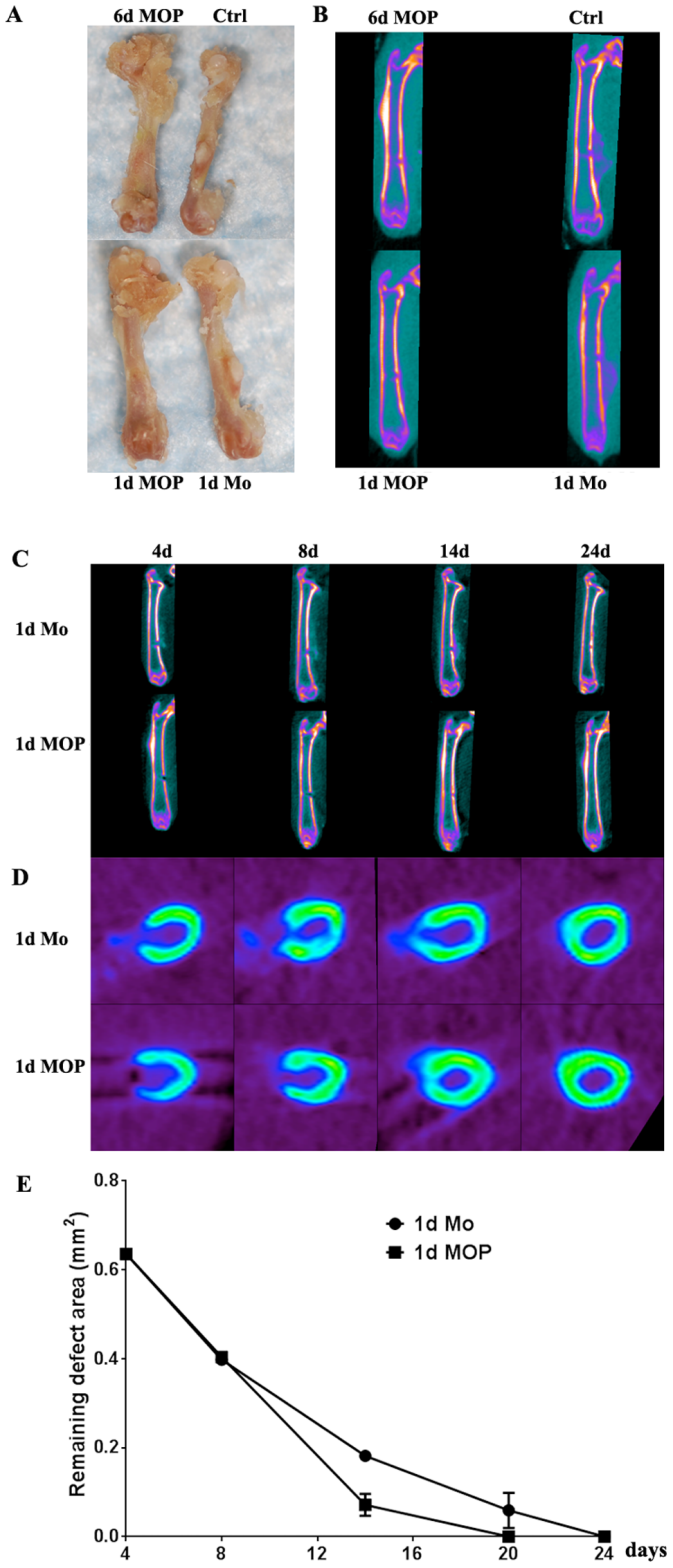
Monoosteophils accelerate cortical bone repair in the drilled-hole bone defect model. NOD/SCID mice were anesthetized with isoflurane, and holes (0.9 mm) were created in the mid-diaphysis of femur. **A–B**. Holes were filled with Matrigel (Ctrl), Matrigel +6 d Monoosteophils (6 d MOP, 3×10^6^ cells), Matrigel+1 d Monoosteophils (1 d MOP, 3×10^6^ cells), or Matrigel +1 d Monocytes (1 d Mo, 3×10^6^ cells). After 14 days, femurs were harvested and observed by visual analysis (**A**) and low resolution µCT (**B**). **C–D**. Holes were filled with Matrigel+1 d Monoosteophils (1 d MOP, 3×10^6^ cells) or Matrigel +1 d Monocytes (1 d Mo, 3×10^6^ cells). Bone repair was monitored by serial µCT using coronal (**C**) and transverse plane imaging (**D**) and quantitated as remaining defect area (mm^2^) (**E**) at days shown in the figure.

In order to examine the bone repair capability of monoosteophils in vivo, drilled hole defects in NOD/SCID mouse femurs were used as a model [34]. Since treatment of monocytes with LL-37 for 24 hours is sufficient to trigger monoosteophil differentiation in vitro (**Figure S7**), both 1 d and 6 d LL-37 generated monoosteophils in Matrigel were applied to drilled hole defects. After application of these cells for 14 days, mouse femurs were analyzed by low-resolution µCT. In Matrigel only (no cells) or Matrigel-monocyte control groups the cortical defect along with bone callus formation was still present at day 14. On the other hand, cortical defects in either the 1 d or 6 d monoosteophil generated groups were almost completely repaired with only trace evidence of bone callus formation (**Figure 6A–B**). These results indicate that 1 d and 6 d LL-37 treated monocytes have the same capacity to accelerate bone repair. Advantages of using the 1 d generated cells include less time of pretreatment and easier cell harvesting since the 1 d cells are loosely adherent compared to strongly adherent 6 d cells.

The kinetics of the bone repair progress was performed using µCT (**Fig. 6C–E**). In the control monocyte group, the critical bone defect was not completely repaired even after twenty-four days. Bone callus formation was obvious at day 4, reached a maximum at day 8, and almost disappeared at day 24. In the LL-37 treated monocyte group, the bone defect was significantly repaired at day 14, similar to the degree of repair as day 24 in the control group, and completely repaired at day 24. Notably, there is no clear bone callus formation during the entire process of bone regeneration in the monoosteophil group.

## Discussion

Bone has a substantial capacity for repair and regeneration in response to injury. Both processes involve a complex integration of cells, growth factors, and extracellular matrix. Although osteoblasts, osteocytes and osteoclasts are resident bone cells, an increasing amount of evidence points to the potential of bone marrow derived hematopoietic cells to engraft into damaged bone and participate in the calcification processes.

Examples of calcifying/bone forming cells from hematopoietic cell lineage are as EPCs [7], MOMPs [8], COCs [9], fibrocytes [10], COP cells [11], monoosteophils [12] and MCCs [13]. They can be grouped into three categories based on their cell surface markers and in vitro generation procedures. The first group are CD34^+^ progenitor cells, COCs [9], [35] and EPCs [36]–[38] have the capacity to differentiate to osteoblastic-like cells [7], [37]. The second group are CD34^+^CD45^+^CD14^+^ peripheral blood cells, including fibrocytes [10], [39], MOMPs [8], and COP [11], isolated from cultured PBMCs and, like MSCs, are able to differentiate into osteoblastic, adipocytic, and myocytic cells. Since CD34 is a marker of hematopoietic stem cells, these cells may be able to differentiate into MSCs, a process called transdifferentiation, followed by differentiation into osteoblast-like cells, adipocytes and chondrocytes. The third group are derived from circulating CD34^−^CD45^+^CD14^+^ cells, or monocytes, and include monoosteophils [12] and MCCs [13], both of which have the capacity to differentiate into novel calcifying/bone forming cells. CD14, a unique lineage marker for human monocytes, is lost on monoosteophils and retained on MCCs. The fact that monocytes may differentiate into a wide range of cellular phenotypes is well known and fits with their role in wound repair [40].

Since monoosteophils are derived from peripheral blood CD34^−^CD45^+^CD14^+^ monocytes and highly express integrin α3, but down-regulate CD14 [12], monoosteophils can be cell surface phenotyped as CD45^+^α3^+^α3β1^+^CD34^−^CD14^−^BAP^−^ cells. Integrin α3β1 is involved in osteoblast-like cell differentiation [41] and is associated with skeletal metastasis of breast and prostate cancer [42], [43], and thus, its up-regulation in monoosteophils may target them to the bone. Furthermore, monoosteophils express different surface markers from MCCs, fibrocytes and MOMPs, suggesting that each may play different roles in remodeling bone.

Given the fact that implantation of monoosteophils in the drilled bone model accelerated bone repair, it is likely that monoosteophils are responsible for at least initiation of the bone repair. Moreover, monoosteophils are also capable of phagocytosis and anti-inflammation (releasing predominant anti-inflammatory cytokines) [12], leading us to hypothesize that monoosteophil treatment may shorten the reactive phase and reparative phase of bone repair, thereby accelerating the bone repair process, as evidenced by lack of callus formation in our study.

Monoosteophil express and release significantly high levels of osteopontin, osteoactivin, cartilage glycoprotein-39, chitinase 1, MMP-9 and -7, CCL-22 and MAPK13. Osteoactivin, cartilage glycoprotein-39, chitinase 1, and MMP-7 distinguish monoosteophils from control monocytes, macrophages, DCs, and osteoclasts. The fact that monocytes from wild type but not *MAPK13* KO mice were able to differentiate into monoosteophils after LL-37 treatment confirms a critical role for this kinase in monoosteophil differentiation. An important question that remains is which receptor(s) are responsible for the activation of the monoosteoophil resulting in the downstream activation of MAPK13, and to a lesser extent Smad1/5/8. Future studies will be directed at the possible connection of MAPK13 signaling.

In a recent review, monoosteophils were hypothesized to be involved in the process of bone maintenance [7], however our present study suggests that their primary role may be in bone repair during wounding, in keeping with the anti-inflammation function of LL-37 which is released during tissue injury. Thus, monoosteophils may potentially be useful in gene and cell therapy protocols to enhance bone formation, remodeling, or regeneration, and in exploring the pathogenesis of ectopic calcification and musculoskeletal diseases, such as atherosclerosis, granuloma calcification and arthritis.

## Methods

### Reagents

LL-37 was synthesized by standard FMOC chemistry, purified by reversed phase HPLC, and the mass verified by nanospray mass spectrometry. EasySep® Human and Mouse Monocyte Enrichment Kit were purchased from StemCell Technologies Inc (Vancouver, Canada), recombinant human GM-CSF, M-CSF, IL-4, and RANKL from ProSpec Tany TechnoGene Ltd (Rehovot, Israel), anti-RUNX2 (Clone 27-K) and anti-Osterix (Clone M-15) from Santa Cruz Biotechnology (Santa Cruz, USA), anti-integrin α3β1 (Clone 2Q993) from US Biological (Swampscott, USA), PE anti-human CD29 (Integrin β1, clone: MAR4), PE anti-human CD18 (Integrin β2, clone 6.7), BioCoat™ Osteologic™ Disc and FITC BrdU/7AAD Flow Kit from BD Biosciences (San Jose, USA). Anti-human integrin β3 (Clone: 25E11), anti-human integrin β4 (Clone: 3E1) and anti-human integrin β5 (Cat#: AB1926-20) were from EHD Millipore (Billerica, MA, USA), PE anti-human CD49a (Integrin α1, clone: TS2/7), FITC anti-human CD49b (Integrin α2, clone: AK-7), Alexa Fluor 488 anti-human CD49c (Integrin α3, Clone ASC-1), PE anti-human CD49d (Integrin α4, clone: 9F10), APC anti-human CD49e (Integrin α5, clone : NK-SAM-1), APC anti-human CD49f (Integrin α6, clone: GoH3), FITC anti-human CD68 (CloneY1/82A) and Alexa Fluor 647 anti-CCR4 (Clone TG6/CCR4) from Biolegend (San Diego, USA), TaqMan array human Pathway Plate from Invitrogen Corp (Grand Island, USA), cell proliferation Dye eFluor670 and eBioscience Fixation/Permeabilization Concentrate and Diluent from eBioscience (San Diego, USA), MMP-7 ELISA Kit from RayBiotech, Inc (Norcross, USA), anti-human p38MAPKα/β/γ (Rabbit), anti-phospho-ERK1/2 (Rabbit), anti-phospho-Smad1/5/8 (Rabbit) and anti-MAPK13 (p38MAPKδ) (Rabbit) from Cell Signaling Technology (Danvers, USA), and EIA kits of Osteopontin, GPNMB/osteoactivin, Cartilage glycoprotein-39 (also called Chitinase 3-like 1), Chitinase 1, MMP-7, and CCL22 from R&D systems (Minneapolis, USA).

### Monocyte Isolation and Differentiation

The use of anonymous discard blood samples without the requirement for informed consents was approved by the City of Hope IRB (IRB # 99132). Peripheral blood mononuclear cells (PBMCs) were isolated from citrated human blood (discard blood from anonymous donors) by centrifugation over Ficoll-Paque Plus (GE healthcare biosciences, Pittsburgh, PA, USA) density gradient. Monocytes were separated using EasySep® Human Monocyte Enrichment Kit from PBMCs. The purified monocytes were stained with anti-CD14-FITC and analyzed using flow cytometry on a FACSCanton II. Monocytes with >95% purity were suspended at 1×10^6^ cells/mL in RPMI 1640 medium supplemented with 10% FBS and treated with 5 µM LL-37 for monoosteophil differentiation. For macrophage differentiation, monocytes were treated with medium only, or 20 ng/mL GM-CSF (GM-Mac), or 50 ng/mL M-CSF (M-Mac) for 6 days. Monocyte-derived DCs were generated with GM-CSF and IL-4 (both at 20 ng/mL). Osteoclasts were differentiated from monocytes in the presence of RANKL and M-CSF (both at 25 ng/mL).

### Scanning Electron Microscopy (SEM) and Energy Dispersive X-ray Spectroscopy (EDS) Analysis

Monocytes at the cell concentration of 1×10^6^ cells/mL in RPMI1640 medium with 10% FBS were incubated with 5 µM LL-37 on BioCoat™ Osteologic™ Discs for 7 weeks in 5% CO_2_ atmosphere and fixed with 2.5% glutaraldehyde in 0.1 M phosphate buffer for SEM. A Thermo Scientific NORAN System 7 EDS (Thermo Fisher Scientific, Madison, WI, USA) incorporated into a SEM system was used for identification of the elemental composition of monoosteophil-formed nodules on Osteologic Discs. Quantitative element maps of built-up structures were determined at high magnification.

### Flow Cytometry

For cell surface staining, cells were washed with PBS, blocked with 10% human serum in PBS, stained with isotype controls or antibodies, washed 3 times with 1% BSA in PBS, and analyzed using FACSCanton II and Flowjo software. For intracellular staining, cells were washed with PBS, fixed and permeabilized with eBioscience Fixation/Permeabilization Concentrate and Diluent, and stained with primary isotype controls or antibodies. After washing with PBS containing 1% BSA and 0.1% saponin, cells were stained with secondary Alexa 488 conjugated antibodies. BrdU/7AAD and Cell proliferation Dye eFluor670 staining were performed according to manufacturers’s procedure.

### Western Blot Analysis

Whole cell extracts of control or LL-37 treated monocyte were prepared using RIPA buffer (50 mM Tris-HCl pH 7.5, 150 mM NaCl, 1% NP40, 0.5% deoxycholate and 0.1% SDS) containing protease inhibitor cocktail (Roche, Basel, Switzerland). Western blotting was performed with anti-human p38MAPKα/β/γ (Rabbit), anti-phospho-ERk1/2 (Rabbit), anti-phospho-Smad1/5/8 (Rabbit), and anti-MAPK13 (p38MAPKδ) (Rabbit).

### Gene Expression Profiling

mRNA was isolated from fresh monocytes (day 0) or 6 day LL-37-differentiated monoosteophils with TRI reagents (Molecular Research Center, Cincinnati, OH). TaqMan Human BMP Pathway Array analysis was performed according to the manufacturers’ company protocol (Invitrogen). Gene expression profiling of Affymetrix Human Genome Array was performed at the City of Hope Microarray Core and microarray data were analyzed using the software Gene Set Enrichment Analysis.

### Enzyme Immunoassay (EIA)

Monocytes were incubated in the absence or presence of LL-37 (5 µM), GM-CSF (20 ng/mL), GM-CSF+IL-4 (both at 20 ng/ml), M-CSF (50 ng/mL) or M-CSF+RANKL (both at 25 ng/mL) for 1 day, 3 day or 6 day. Quantification of soluble Osteopontin, Osteoactivin/GPNMB, cartilage glycoprotein 39, Chitinase 1, MMP-7, and CCL22 was performed using commercially available EIA kits (R&D systems, Minneapolis, MN).

### Immunofluorescence Staining

Monocytes were incubated in the presence or absence of 5 µM LL-37 for 6 days, washed twice with cold PBS, fixed and permeabilized with eBioscience Fixation/Permeabilization reagent. Unspecific binding was blocked with 10% human serum before staining with a monoclonal anti-RunX2 or anti-Osterix antibody at 4°C overnight. Cells were incubated with a secondary Alexa Fluor 488 labeled donkey anti-mouse-Ig antibody (Invitrogen) for 45 min. Images were obtained using immunofluorescence microscopy.

### LL-37 Triggers Monocyte Differentiation to Monoosteophil

Monocytes were treated with or without 5 µM LL-37 for different time points, and replaced with fresh media without LL-37 for an additional 6 days. Cell morphology were recorded with Leica DMI 3000B (Leica Microsystems Inc, Bonnockburn, IL60015) inverted microscope.

### Mice and Surgery

All animal experiments were approved by the City of Hope Institutional Animal Care and Use committee (IACUC), IACUC protocol number 09028. NOD/SCID male mice purchased from The Jackson Laboratory (Bar Harbor, Maine) were kept at 22–25°C under a 12-hour light/dark cycle. Fourteen week old mice were anesthesized with isoflurane and holes (0.9 mm) were drilled in the femur using an electric drill (Harvard Apparatus, Cambridge, MA) at 10,000 rpm [34]. The operating field was frequently irrigated with saline to avoid thermal necrosis. In the mid-diaphysis, through-and-through perforations disrupted cortical, periosteal, and endosteal surfaces and extended 0.3–0.5 mm into the marrow; but the drill did not reach the opposite cortical wall. Groups (6–10 animals per group) were Matrigel only (no cells, 100 µL Matrigel), Matrigel +1 d monocytes (3 ×10^6^) and Matrigel+1 d or 6 d monoosteophils (3 × 10^6^). Buprenorphine (0.05 mg/kg, s.c) was injected at the end of surgical procedure to allevaite the pain and continued once per day until 6 days and animals also received tetracycline antibiotic through water (0.5 mg/ml). The animals were monitored daily over the course of 2 weeks for any signs of infection or distress. At the end of the observeration period, animals were euthanized with CO_2_ until cessation of breathing was observed.

*MAPK13* KO mice were kindly supplied by Dr. James Simon Campbell Arthur, Divesion of Signal Transduction Therapy, University of Dundee, United Kingdom. Generation of *MAPK*13 KO mice were described previously [44], [45]. Monocytes from bone marrow were isolated using EasySep™ Mouse Monocyte Enrichment Kit. Monocyte purity was checked using anti-CD11b and anti-Ly6C (purity >95%), cultured at 1×10^6^ cells/mL in RPMI 1640 medium supplemented with 10% FBS, and treated with 5 µM LL-37 for monoosteophil differentiation.

### Micro-computed Tomography (µCT)

At each time point 4 d, 8 d, 14 d, and 24 d after surgery, femurs were examined using Siemens MicroCAT II Ultra Hi-Res (Siemens Medical Solutions, Knoxville, TN). Reconstruction of the diaphyseal region was performed following correction of the rotation center and calibration of the mineral density. Bone analysis was performed using MicroDicom software.

### Statistical Analysis

Assay results were expressed as means ±SEM and unpaired Student's t-tests were used for comparisons. All p-values are two-sided. Data were analyzed with GraphPad Prism software (version 5.0, GraphPad Software, San Diego, CA, USA).

## Supporting Information

Figure S1 **Surface integrin α and β expression in monoosteophil differentiation.** Monocytes were incubated in the absence or presences of LL-37 (5 µM), GM-CSF (20 ng/mL), M-CSF (50 ng/mL) or M-CSF+RANKL (both at 25 ng/mL) for 6 days. Surface staining of integrin α1-6 and β1-5 were analyzed using flow cytometry. Data shown were from at least three independent experiments.
(PDF)

Figure S2 **Time course of expression of integrin α3 and α3β1 during monoosteophil differentiation.** Monocytes were incubated in the presence of LL-37 (5 µM), GM-CSF (20 ng/mL) or M-CSF (50 ng/mL) for 1 or 3 days. Surface staining of integrin α3 and α3β1 were analyzed using flow cytometry. Data shown were from at least three independent experiments.
(TIF)

Figure S3 **CD68 and CCR4 expression in monoosteophils and macrophages.** Monocytes were incubated in the absences or presence of LL-37 (5 µM), GM-CSF (20 ng/mL) or M-CSF (50 ng/mL) for 6 day. Surface staining of CD68 and CCR4 were analyzed using flow cytometry. Data shown were from at least three independent experiments.
(TIF)

Figure S4 **p38MAPKα/β/γ, ERK, RunX2 and Osterix signaling in monoosteophils.** Monocytes were incubated in the absence or presence of LL-37 (5 µM). Cells were harvested on day 6 and p38MAPKα/β/γ, pERK42/44, RunX2 and osterix were analyzed using western blot or fluorescence microscopy. Data shown were from three independent experiments.
(TIF)

Figure S5 **mRNA levels of targeted genes during monoosteophil differentiation.** Monocytes were treated with 5 µM LL-37 and harvested at different time points. Gene expression of treated cells in comparison with fresh monocytes was performed using gene chip analysis. Targeted genes were shown as fold change.
(TIF)

Figure S6 **Intergrin α3 and α6, CD68, and alkaline phosphatase expression on the surface of 6 d monoosteophils from wild type mice.** Negatively isolated monocytes from bone marrow cells of wild type mice were incubated in the presence of CRAMP (mouse LL-37, 5 µM) or mouse GM-CSF (20 ng/mL) for 6 days. Surface staining of integrin α3 and α6, CD68 and alkaline phosphatase were analyzed using flow cytometry. Data shown were from three independent experiments.
(TIF)

Figure S7 **LL-37 triggers monocyte differentiation into monoosteophils.** Monocytes at the concentration of 1×10^6^/mL were incubated in RPMI 1460 medium with 10% FBS in the absence or presence of LL-37 for different time points and cell morphology on day 6 was observed by phase contrast microscopy (magnification 200×). Data shown were from at least three independent experiments.
(TIF)

Table S1 **Gene expression of monoosteophils using a Human BMP Pathway Array.** Genes (59) in the BMP array were analyzed for fold change in freshly isolated monocytes vs monocytes treated with LL-37 for 6 days.
(DOCX)

Table S2 **Gene expression of monoosteophils using Affymetrix Human Genome Array.** Freshly isolated monocytes vs monocytes treated with LL-37 for 6 days were analyzed using the Affymetrix gene chip analysis. Genes with a fold change >100 plus those associated with osteoblast, osteocyte and osteoclast were reported.
(DOCX)
