# Supplementary figures and images for: Acceleration of Bone Repair in NOD/SCID Mice by Human Monoosteophils, Novel LL-37-Activated Monocytes
Source: PLoS One. 2013 Jul 3;8(7):e67649. doi: 10.1371/journal.pone.0067649 (PMC3701041; doi:10.1371/journal.pone.0067649)

Fig. S1

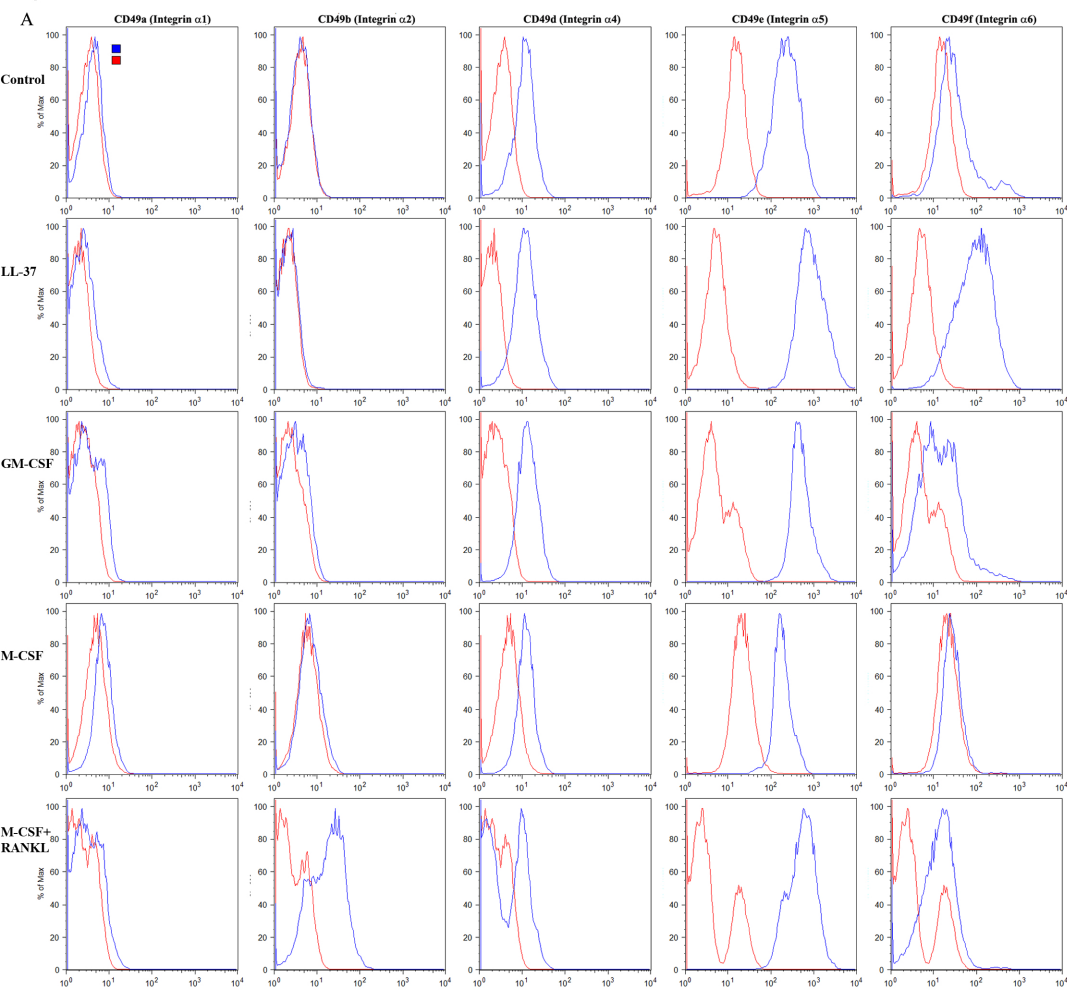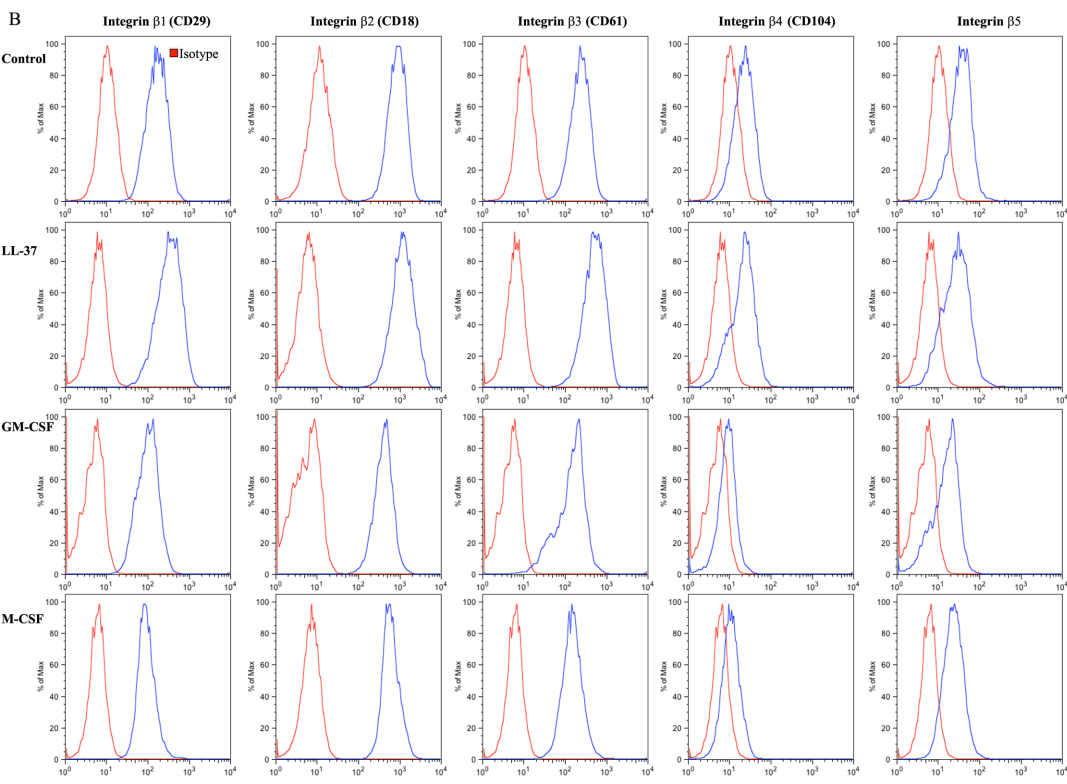

Supplement: Figure S1 — Surface integrin α and β expression in monoosteophil differentiation. Monocytes were incubated in the absence or presences of LL-37 (5 µM), GM-CSF (20 ng/mL), M-CSF (50 ng/mL) or M-CSF+RANKL (both at 25 ng/mL) for 6 days. Surface staining of integrin α1-6 and β1-5 were analyzed using flow cytometry. Data shown were from at least three independent experiments. (PDF) [file pone.0067649.s001.pdf]

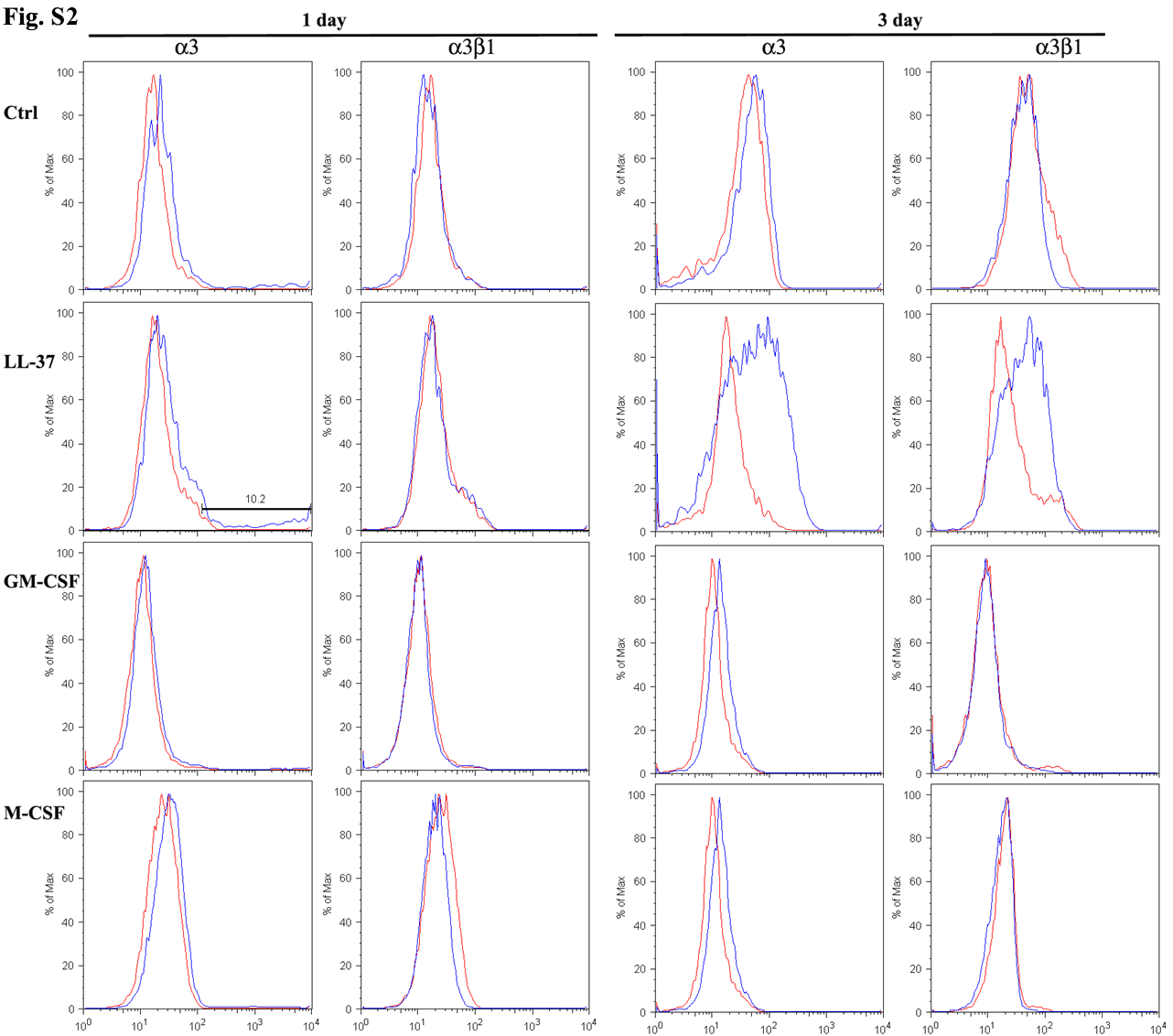

Supplement: Figure S2 — Time course of expression of integrin α3 and α3β1 during monoosteophil differentiation. Monocytes were incubated in the presence of LL-37 (5 µM), GM-CSF (20 ng/mL) or M-CSF (50 ng/mL) for 1 or 3 days. Surface staining of integrin α3 and α3β1 were analyzed using flow cytometry. Data shown were from at least three independent experiments. (TIF) [file pone.0067649.s002.tif]

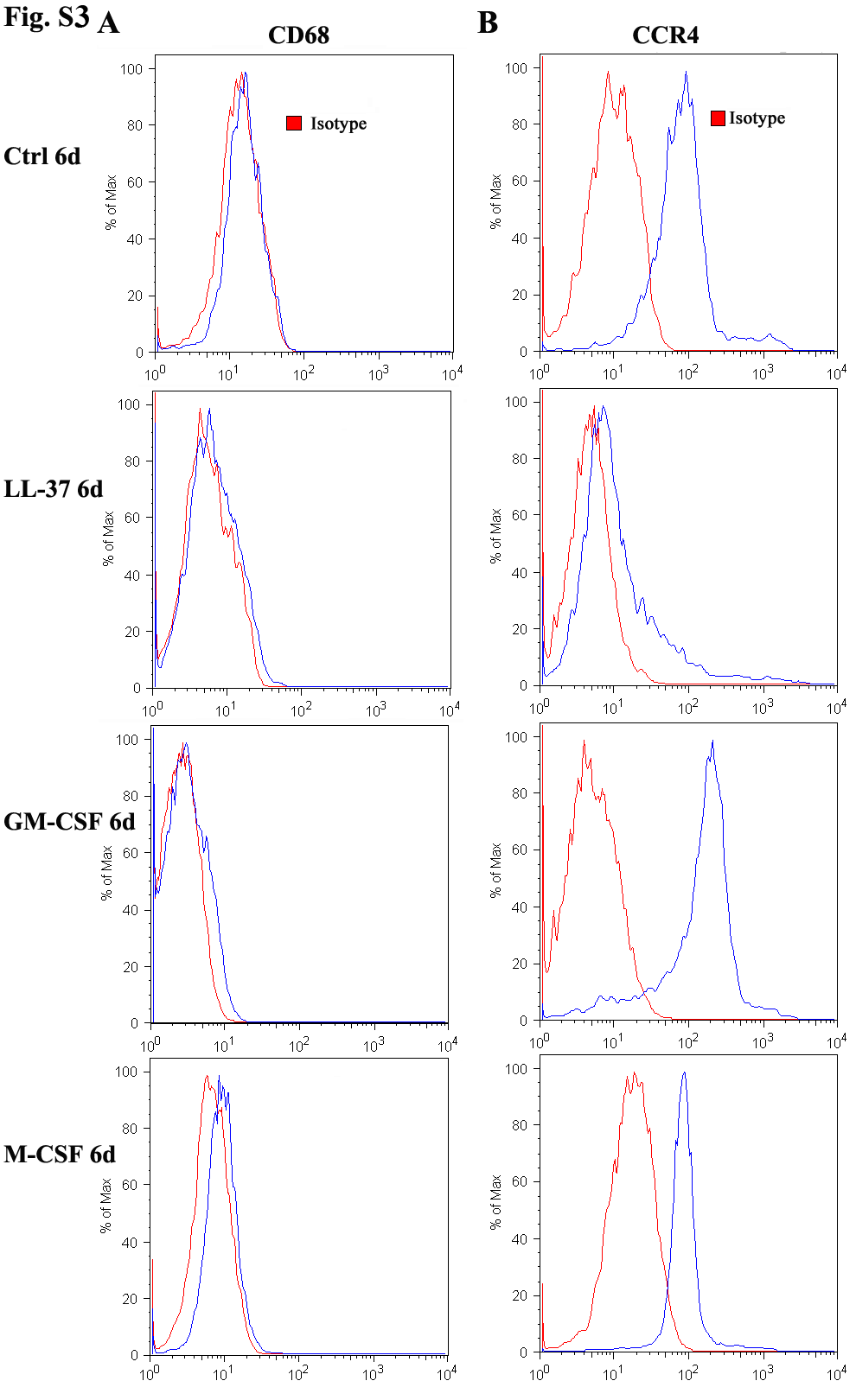

Supplement: Figure S3 — CD68 and CCR4 expression in monoosteophils and macrophages. Monocytes were incubated in the absences or presence of LL-37 (5 µM), GM-CSF (20 ng/mL) or M-CSF (50 ng/mL) for 6 day. Surface staining of CD68 and CCR4 were analyzed using flow cytometry. Data shown were from at least three independent experiments. (TIF) [file pone.0067649.s003.tif]

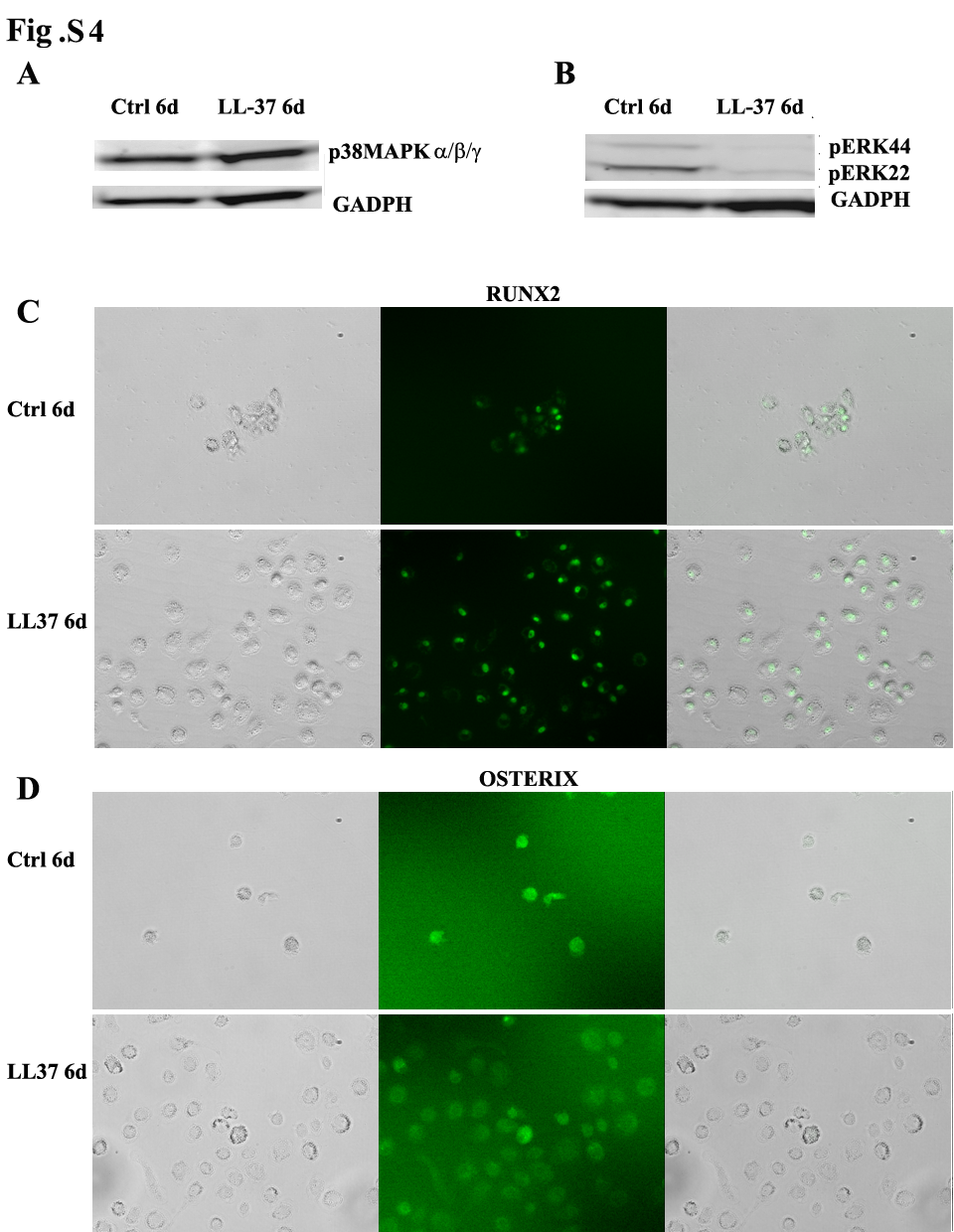

Supplement: Figure S4 — p38MAPKα/β/γ, ERK, RunX2 and Osterix signaling in monoosteophils. Monocytes were incubated in the absence or presence of LL-37 (5 µM). Cells were harvested on day 6 and p38MAPKα/β/γ, pERK42/44, RunX2 and osterix were analyzed using western blot or fluorescence microscopy. Data shown were from three independent experiments. (TIF) [file pone.0067649.s004.tif]

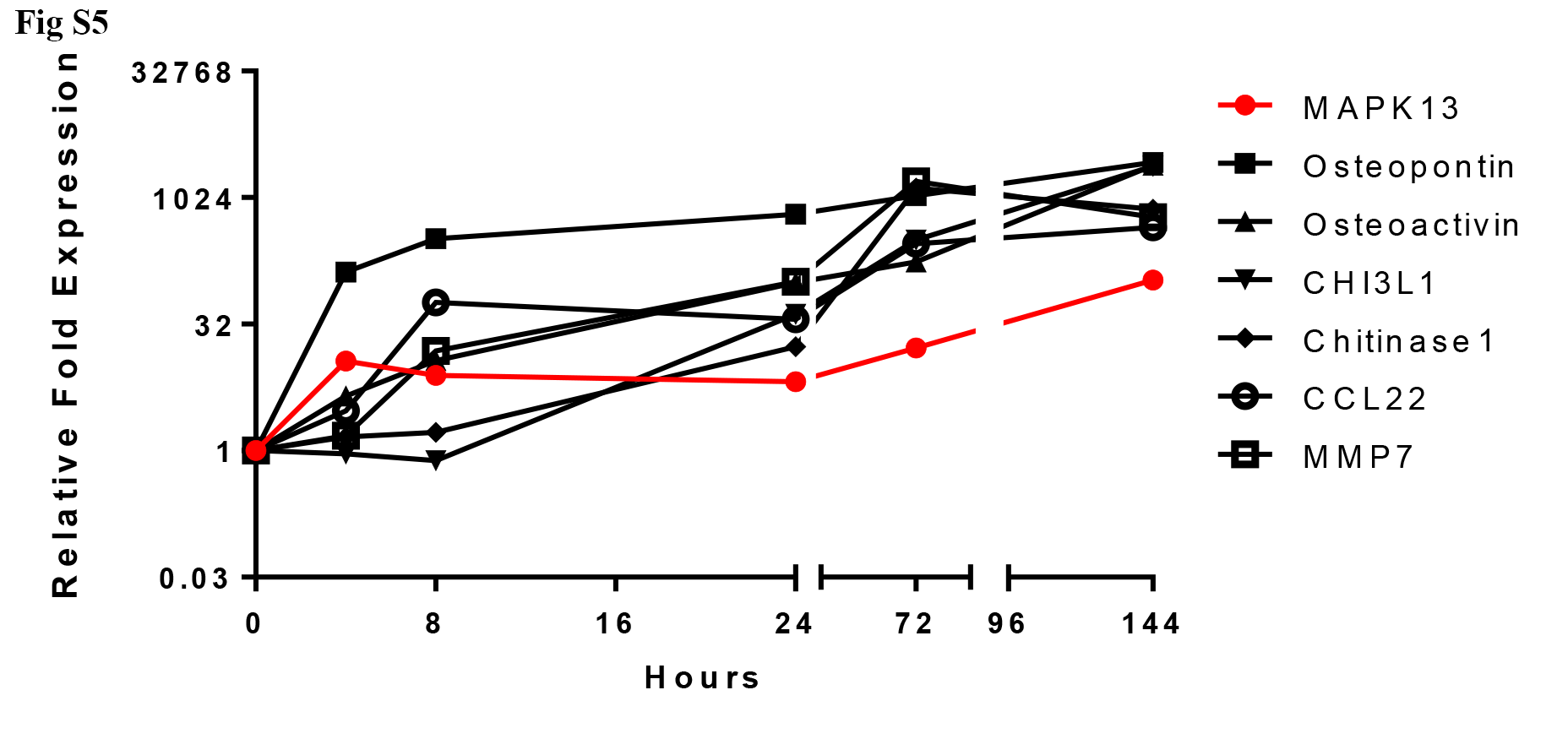

Supplement: Figure S5 — mRNA levels of targeted genes during monoosteophil differentiation. Monocytes were treated with 5 µM LL-37 and harvested at different time points. Gene expression of treated cells in comparison with fresh monocytes was performed using gene chip analysis. Targeted genes were shown as fold change. (TIF) [file pone.0067649.s005.tif]

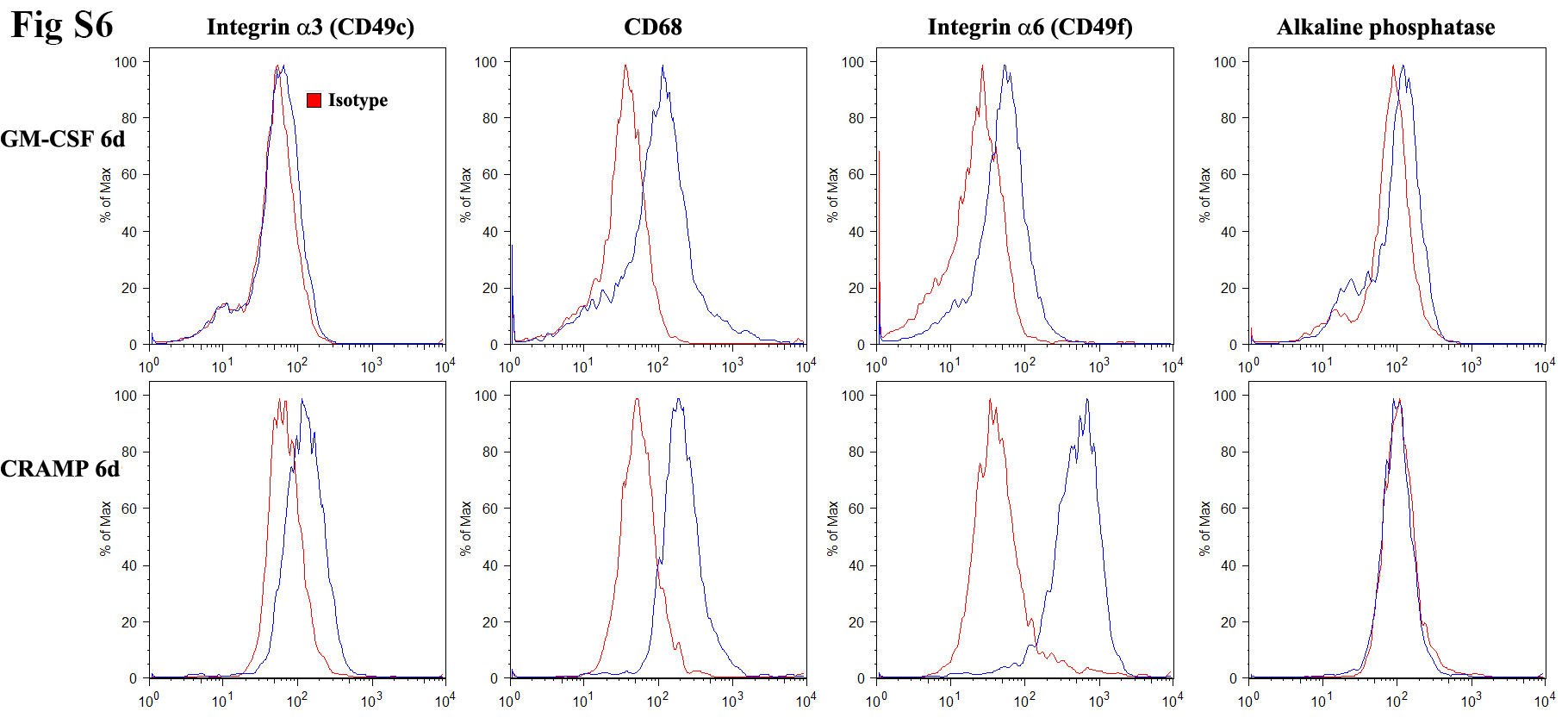

Supplement: Figure S6 — Intergrin α3 and α6, CD68, and alkaline phosphatase expression on the surface of 6 d monoosteophils from wild type mice. Negatively isolated monocytes from bone marrow cells of wild type mice were incubated in the presence of CRAMP (mouse LL-37, 5 µM) or mouse GM-CSF (20 ng/mL) for 6 days. Surface staining of integrin α3 and α6, CD68 and alkaline phosphatase were analyzed using flow cytometry. Data shown were from three independent experiments. (TIF) [file pone.0067649.s006.tif]

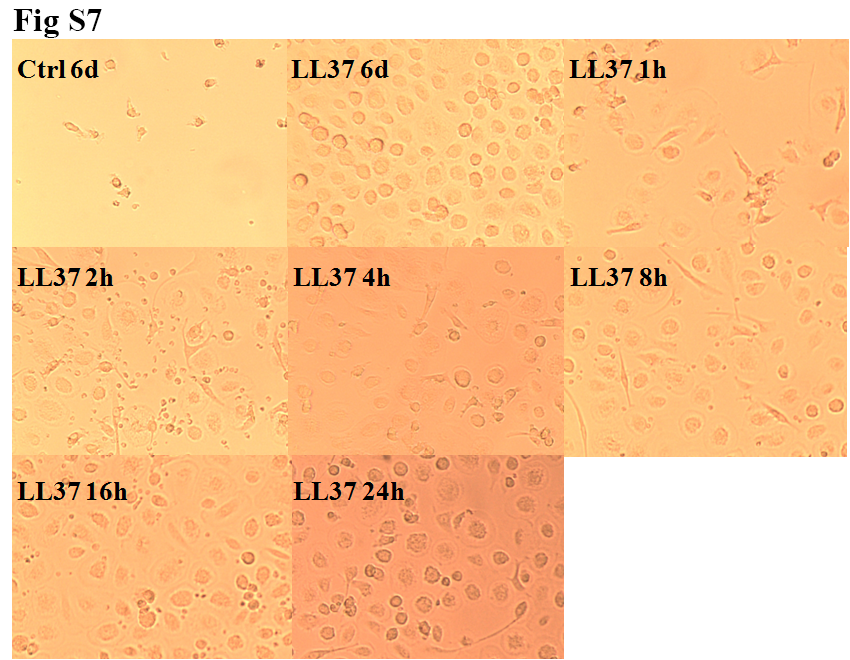

Supplement: Figure S7 — LL-37 triggers monocyte differentiation into monoosteophils. Monocytes at the concentration of 1×10^6^/mL were incubated in RPMI 1460 medium with 10% FBS in the absence or presence of LL-37 for different time points and cell morphology on day 6 was observed by phase contrast microscopy (magnification 200×). Data shown were from at least three independent experiments. (TIF) [file pone.0067649.s007.tif]
