# Supplementary material for: Acceleration of Bone Repair in NOD/SCID Mice by Human Monoosteophils, Novel LL-37-Activated Monocytes
Source: PLoS One. 2013 Jul 3;8(7):e67649. doi: 10.1371/journal.pone.0067649 (PMC3701041; doi:10.1371/journal.pone.0067649)
Supplement: Table S1 — Gene expression of monoosteophils using a Human BMP Pathway Array. Genes (59) in the BMP array were analyzed for fold change in freshly isolated monocytes vs monocytes treated with LL-37 for 6 days. (DOCX) [file pone.0067649.s008.docx]

**Table S1. Gene expression of monoosteophils using a Human BMP Pathway Array ^a^**

| **Target genes** | **Fold change** |  | **Target genes** | **Fold change** |
| --- | --- | --- | --- | --- |
| *MAPK13* | 18.00 |  | *CREB1* | 0.36 |
| *PRKACB* | 4.31 |  | *NFKB2* | 0.36 |
| *BMPR1A* | 2.96 |  | *MAPK8* | 0.33 |
| *TFRC* | 2.73 |  | *SMAD4* | 0.32 |
| *MAPK11* | 2.12 |  | *RELB* | 0.29 |
| *MAPK12* | 2.05 |  | *JUN* | 0.29 |
| *BMP2* | 1.93 |  | *PRKACA* | 0.26 |
| *CREB3* | 1.49 |  | *BMPR2* | 0.26 |
| *ACTB* | 1.46 |  | *MAPK14* | 0.24 |
| *SMAD6* | 1.23 |  | *ATF4* | 0.24 |
| *MAPK10* | 1.08 |  | *B2M* | 0.21 |
| *ACVR2B* | 0.90 |  | *CACNA1F* | 0.18 |
| *MAPK9* | 0.81 |  | *REL* | 0.16 |
| *MAP3K7IP2* | 0.75 |  | *NFKB1* | 0.16 |
| *PRKAR1A* | 0.74 |  | *CACNB3* | 0.15 |
| *CREB3L4* | 0.73 |  | *EP300* | 0.14 |
| *CACNA2D4* | 0.68 |  | *CAMK4* | 0.13 |
| *ACVR1* | 0.67 |  | *PRKAR2B* | 0.11 |
| *SMAD7* | 0.62 |  | *CACNB1* | 0.08 |
| *PGK1* | 0.59 |  | *CREBBP* | 0.07 |
| *PRKAR1B* | 0.58 |  | *CACNA1D* | 0.05 |
| *ATF2* | 0.58 |  | *CACNA2D3* | 0.02 |
| *OAZ1* | 0.57 |  | *BMP4* | 0.02 |
| *RPLP0* | 0.56 |  | *PRKACG* | 0.01 |
| *MAP3K7* | 0.53 |  | *GATA3* | 0.01 |
| *HMBS* | 0.52 |  | *CACNB4* | 0.01 |
| *MAP3K7IP1* | 0.51 |  | *FOS* | 0.01 |
| *RELA* | 0.48 |  | *GATA2* | 0.008 |
| *SMAD5* | 0.46 |  | *GATA1* | 0.006 |
| *SMAD1* | 0.38 |  |  |  |

^a^ Fold change calculated from d0 to d6.
