# Supplementary material for: Acceleration of Bone Repair in NOD/SCID Mice by Human Monoosteophils, Novel LL-37-Activated Monocytes
Source: PLoS One. 2013 Jul 3;8(7):e67649. doi: 10.1371/journal.pone.0067649 (PMC3701041; doi:10.1371/journal.pone.0067649)
Supplement: Table S2 — Gene expression of monoosteophils using Affymetrix Human Genome Array. Freshly isolated monocytes vs monocytes treated with LL-37 for 6 days were analyzed using the Affymetrix gene chip analysis. Genes with a fold change >100 plus those associated with osteoblast, osteocyte and osteoclast were reported. (DOCX) [file pone.0067649.s009.docx]

**Table S2 Gene expression of monoosteophils using Affymetrix Human Genome Array ^a^**

| **Gene symbol** | **Gene name** | **Fold change** | | **Bone cell genotype^b^** |
| --- | --- | --- | --- | --- |
| *SPP1* | Secreted phosphoprotein 1 (osteopontin) | | 2,694 | OB |
| *GPNMB* | Glycoprotein (transmembrane) nmb | | 2,477 |  |
| *CHI3L1* | Chitinase 3-like 1 (cartilage glycoprotein-39) | | 2,409 |  |
| *LPL* | Lipoprotein lipase | | 1,001 |  |
| *APOE* | Apolipoprotein E | | 778 |  |
| *CHIT1* | Chitinase 1 (chitotriosidase) | | 743 |  |
| *MMP9* | Matrix metallopeptidase 9 (gelatinase B) | | 680 | OC |
| *FABP4* | Fatty acid binding protein 4, adipocyte | | 637 |  |
| *GAL* | Galanin prepropeptide | | 620 |  |
| *APOC1* | Apolipoprotein C-I | | 614 |  |
| *MMP7* | Matrix metallopeptidase 7 (matrilysin, uterine) | | 599 | OC |
| *TIMP3* | TIMP metallopeptidase inhibitor 3 | | 584 |  |
| *CCL22* | Chemokine (C-C motif) ligand 22 | | 451 |  |
| *MREG* | Melanoregulin | | 431 |  |
| *GPC4* | Glypican 4 | | 389 |  |
| *SDC2* | Syndecan 2 | | 355 |  |
| *A2M* | Alpha-2-macroglobulin | | 333 |  |
| *SUCNR1* | Succinate receptor 1 | | 309 |  |
| *C15orf48* | Chromosome 15 open reading frame 48 | | 275 |  |
| *NCAPH* | Non-SMC condensin I complex, subunit H | | 268 |  |
| *TREM2* | Triggering receptor expressed on myeloid cells 2 | | 255 |  |
| *UCHL1* | Ubiquitin carboxyl-terminal esterase L1 | | 250 |  |
| *TM7SF4* | Transmembrane 7 superfamily member 4 (DC-STAMP) | | 224 |  |
| *FABP3* | Fatty acid binding protein 3 | | 209 |  |
| *ATP6V0D2* | ATPase, H+ transporting, lysosomal 38kDa, V0 subunit d2 | | 184 | OC |
| *EMP1* | Epithelial membrane protein 1 | | 183 |  |
| *C11orf45* | Chromosome 11 open reading frame 45 | | 180 |  |
| *AFAP1L1* | Actin filament associated protein 1-like 1 | | 171 |  |
| *BHLHE41* | Basic helix-loop-helix family, member e41 | | 171 |  |
| *HAMP* | Hepcidin antimicrobial peptide | | 155 |  |
| *TGM2* | Transglutaminase 2 | | 146 |  |
| *DNASE2B* | Deoxyribonuclease II beta | | 144 |  |
| *NRIP3* | Nuclear receptor interacting protein 3 | | 142 |  |
| *CRABP2* | Cellular retinoic acid binding protein 2 | | 138 |  |
| *PSD3* | Pleckstrin and Sec7 domain containing 3 | | 131 |  |
| *GM2A* | GM2 ganglioside activator | | 122 |  |
| *PPARG* | Peroxisome proliferator-activated receptor gamma | | 116 |  |
| *PPAP2B* | Phosphatidic acid phosphatase type 2B | | 115 |  |
| *MSR1* | Macrophage scavenger receptor 1 | | 113 |  |
| *FAIM3* | Fas apoptotic inhibitory molecule 3 | | 107 |  |
| *SCD* | Stearoyl-CoA desaturase (delta-9-desaturase) | | 107 |  |
| *MAPK13* | Mitogen-activated protein kinase 13 (p38MAPKδ) | | 106 |  |
| *ADAMDEC1* | ADAM-like, decysin 1 | | 100 |  |
| *SPARC* | Secreted protein, acidic, cysteine-rich (osteonectin) | | 10 | OB |
| *ALPL* | Alkaline phosphatase, liver/bone/kidney | | 1 | OB |
| *BGLAP* | Osteocalcin | | -1 | OB |
| *IBSP* | Integrin-binding sialoprotein (BSP II) | | 1 | OB |
| *TNFSF11* | Tumor necrosis factor (ligand) superfamily, (RANKL) | | 1 | OB |
| *RUNX2* | Runt-related transcription factor 2 | | 1 | OB |
| *SP7* | Sp7 transcription factor (Osterix) | | 1 | OB |
| *SMAD1* | SMAD family member 1 | | -1 | OB |
| *SMAD2* | SMAD family member 2 | | 2 | OB |
| *SMAD3* | SMAD family member 3 | | -1 | OB |
| *SMAD4* | SMAD family member 4 | | -1 | OB |
| *SMAD5* | SMAD family member 5 | | -1 | OB |
| *COL1A1* | Collagen, type I, alpha 1 | | 1 | OB |
| *EPHB4* | EPH receptor B4 | | -1 | OB |
| *MAPK11* | Mitogen-activated protein kinase 11 (p38MAPKβ) | | 1 | OB |
| *MAPK12* | Mitogen-activated protein kinase 11 (p38MAPKγ) | | 1 | OB |
| *MAPK14* | Mitogen-activated protein kinase 14 (p38MAPKα) | | -2 | OB |
| *MMP14* | Matrix metallopeptidase 14 (membrane-inserted) | | 4 | OCE |
| *DSTN* | Destrin (actin depolymerizing factor) | | 4 | OCE |
| *PDPN* | Podoplanin | | 1 | OCE |
| *MEPE* | Matrix extracellular phosphoglycoprotein | | 1 | OCE |
| *PHEX* | Phosphate regulating endopeptidase homolog, X-linked | | 1 | OCE |
| *DMP1* | Dentin matrix acidic phosphoprotein 1 | | 1 | OCE |
| *CAPG* | Capping protein (actin filament), gelsolin-like | | 8 | OCE |
| *SOSTDC1* | Sclerostin domain containing 1 | | -1 | OCE |
| *SOST* | Sclerosteosis | | 1 | OCE |
| *FGF23* | Fibroblast growth factor 23 | | 1 | OCE |
| *HYOU1* | Hypoxia up-regulated 1 | | 1 | OCE |
| *ACP5* | Acid phosphatase 5, tartrate resistant | | 59 | OC |
| *TNFRSF11A* | Tumor necrosis factor receptor superfamily,(RANK) | | 11 | OC |
| *CTSK* | Cathepsin K | | 15 | OC |
| *CA2* | Carbonic anhydrase II | | 13 | OC |
| *ITGB3* | Integrin, beta 3 (antigen CD61) | | 1 | OC |
| *ITGB3BP* | Integrin beta 3 binding protein (beta3-endonexin) | | 2 | OC |
| *EFNB2* | Ephrin-B2 | | 1 | OC |
|  |  |  | |  |

^a^ Fold change calculated from d0 to d6 for fold ≥100, plus selected bone genes.

^b^ OB: osteoblast-like genes [^1^](#_ENREF_1); OC: osteoclast-like genes [^2^](#_ENREF_2); OCE: osteocyte-like genes [^3^](#_ENREF_3).

^1^ Miron, R.J. & Zhang, Y.F. Osteoinduction: a review of old concepts with new standards. Journal of dental research 91, 736-744 (2012).

^2^ Boyle, W.J., Simonet, W.S. & Lacey, D.L. Osteoclast differentiation and activation. Nature 423, 337-342 (2003).

^3^ Bonewald, L.F. The amazing osteocyte. J Bone Miner Res 26, 229-238 (2011).
